# Supplementary material for: The Caulobacter NtrB-NtrC two-component system bridges nitrogen assimilation and cell development
Source: bioRxiv. 2023 Aug 31:2023.06.06.543975. Originally published 2023 Jun 7. Preprint. [Version 2] doi: 10.1101/2023.06.06.543975 (PMC10274813; doi:10.1101/2023.06.06.543975)
Supplement: Supplement 5 [file NIHPP2023.06.06.543975v2-supplement-5.pdf]

bioRxiv preprint doi: https://doi.org/10.1101/2023.06.06.543975; this version posted August 31, 2023. The copyright holder for this preprint (which was not certified by peer review) is the author/funder, who has granted bioRxiv a license to display the preprint in perpetuity. It is made available under aCC-BY 4.0 International license.

Supplemental Figures

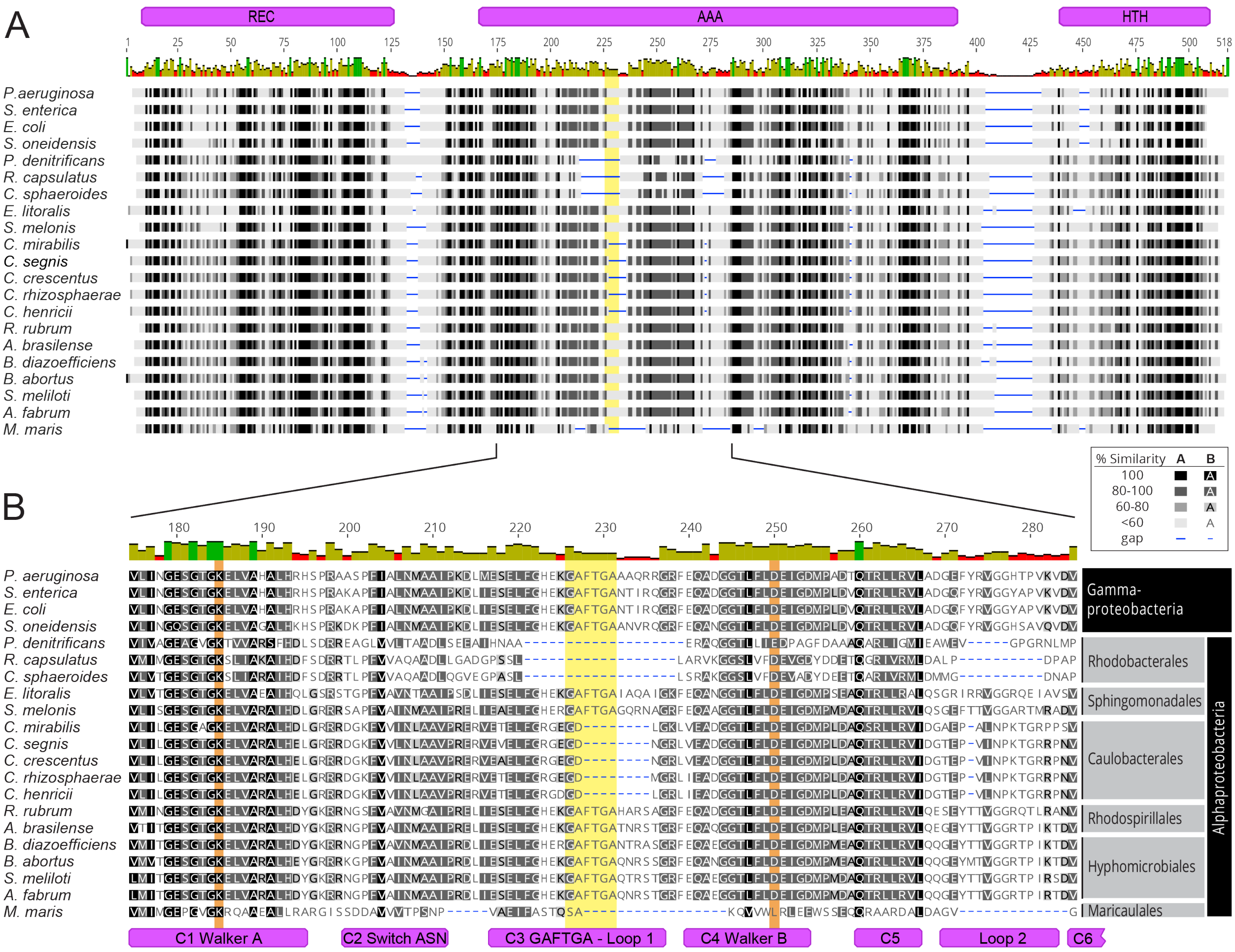

Figure S1. Multiple sequence alignment revealing conserved and distinctive characteristics of NtrC/GlnG sequences. (A) Overview of full length NtrC/GlnG protein sequence alignment. Residue numbers indicate alignment positions. (B) Highlight of the sequences within the AAA+ domain including the Walker A, Walker B and GAFTGA loop regions. Alignment was performed using Clustal Omega {McWilliam, 2013 #77}, and similarity scores were calculated based on the Blosum62 matrix. In the alignment, gaps are denoted by blue lines. The L1 loop containing the GAFTGA motif is highlighted in yellow. Key ATP binding and ATP hydrolysis residues described in the main text are highlighted in orange. The domains are described following the nomenclature outlined in Bush and Dixon {Bush, 2012 #16}. Species are indicated at the left of aligned sequences. Full organism names and genbank accessions for this analysis in the order presented in the alignment are: *Pseudomonas aeruginosa* PAO1 (NZ_CP053028), *Salmonella enterica* subsp. enterica serovar Typhimurium str. 798 (NC_017046), *Escherichia coli* str. K-12 substr. MG1655 (NZ_CP027060), *Shewanella oneidensis* MR-1 (NC_004347), *Paracoccus denitrificans* PD1222 (NC_008687), *Rhodobacter capsulatus* SB 1003 (NC_014034), *Cereibacter sphaeroides* 2.4.1 (formerly *Rhodobacter sphaeroides*; NC_007493), *Erythrobacter litoralis* DSM 8509 (NZ_CP017057), *Sphingomonas melonis* TY (NZ_CP017578), *Caulobacter mirabilis* (NZ_CP024201), *Caulobacter segnis* ATCC 21756 (NC_014100), *Caulobacter crescentus* NA1000 (NC_011916), *Caulobacter rhizosphaerae* (NZ_CP048815), *Caulobacter henricii* (NZ_CP013002), *Rhodospirillum rubrum* ATCC 11170 (NC_007643), *Azospirillum brasilense* (NZ_CP012915), *Bradyrhizobium diazoefficiens* USDA 110 (formerly B. japonicum; NC_004463), *Brucella abortus* 2308 (NC_007618), *Sinorhizobium meliloti* 1021 (NC_003037), *Agrobacterium fabrum* str. C58 (formerly A. tumefaciens; NC_003062), *Maricaulis maris* MCS10 (NC_008347). We note that within the Caulobacterales, we also searched the following complete genomes: *Asticcacaulis excentricus* CB 48 (NC_014816), *Brevundimonas diminuta* (NZ_CP021995), *Brevundimonas nasdae* (NZ_CP080036) and *Brevundimonas subvibrioides* ATCC 15264 (NC_014375). We found that genomes from the genera *Asticcacaulis* and *Brevundimonas* lack *ntrC* and possess only *ntrX* at the *ntr* locus. NtrX appears to be specific to the Alphaproteobacteria and lacks the classical GAFTGA sequence motif; NtrX sequences exhibit a shorter motif (EG**EG---G**xxK/R) where the dashes are gaps compared to the motif found in most enhancer binding proteins (EK**GAFTGA**xxK/R).

bioRxiv preprint doi: https://doi.org/10.1101/2023.06.06.543975; this version posted August 31, 2023. The copyright holder for this preprint (which was not certified by peer review) is the author/funder, who has granted bioRxiv a license to display the preprint in perpetuity. It is made available under aCC-BY 4.0 International license.

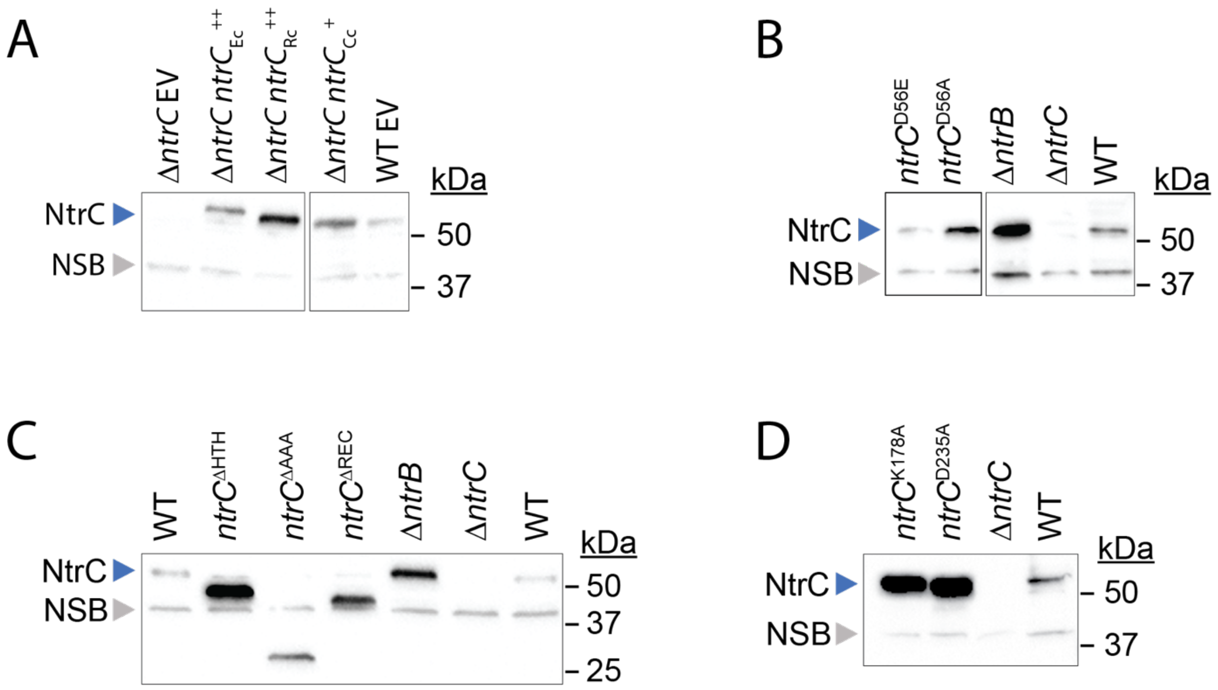

Figure S2. Western blot to assess steady state levels of *Caulobacter* NtrC alleles, and *E. coli* and *R. capsulatus* NtrC expressed in *Caulobacter*. In all panels, wild-type *Caulobacter* NtrC is marked by the blue arrow (~52 kDa); the non-specific band (NSB) serving as a loading control is marked with a gray arrow. Panel (A) shows an α-NtrC western blot of lysates from *Caulobacter* grown to stationary phase in PYE complex medium supplemented with 0.15% xylose. Displayed are WT and Δ*ntrC* carrying either an empty vector (EV) or expression vectors containing *Caulobacter ntrC* under the control of its native promoter (*ntrC*$_{Cc}^{+}$), or *E. coli* or *R. capsulatus ntrC* under the control of a xylose-inducible promoter (*ntrC*$_{Ec}^{++}$ or *ntrC*$_{Rc}^{++}$). Panels (B-C) present an α-NtrC western blot of lysates WT, Δ*ntrC*, Δ*ntrB*, *ntrC*^{D56A}, *ntrC*^{D56E}, *ntrC*$^{\Delta REC}$ (residues deleted: 17-125), *ntrC*$^{\Delta AAA}$ (residues deleted: 159-363), and *ntrC*$^{\Delta HTH}$ (residues deleted: 423-462) grown to logarithmic phase in PYE. The predicted molecular weights of NtrC$^{\Delta REC}$, NtrC$^{\Delta AAA}$, and NtrC$^{\Delta HTH}$ are approximately 41 kDa, 30 kDa, and 48 kDa, respectively. Panel (D) shows an α-NtrC western blot of lysates WT, Δ*ntrC*, *ntrC*^{K178A} (Walker A mutant), and *ntrC*^{D235A} (Walker B mutant) grown to stationary phase in PYE.

bioRxiv preprint doi: https://doi.org/10.1101/2023.06.06.543975; this version posted August 31, 2023. The copyright holder for this preprint (which was not certified by peer review) is the author/funder, who has granted bioRxiv a license to display the preprint in perpetuity. It is made available under aCC-BY 4.0 International license.

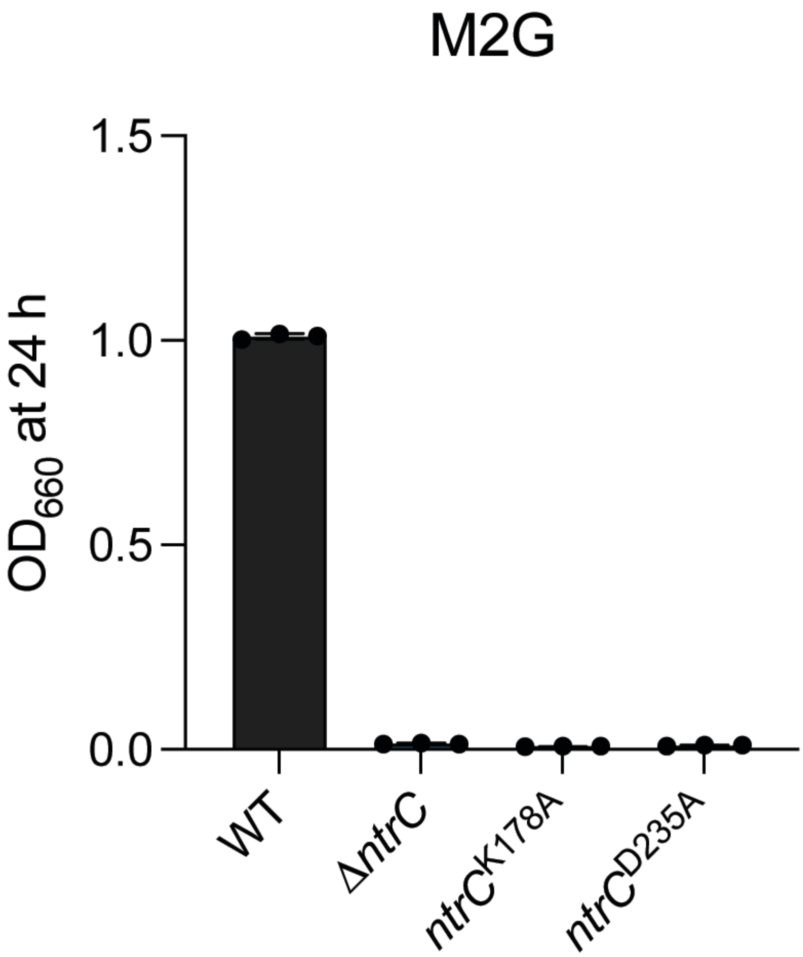

Figure S3. Conserved residues of Walker A and Walker B motifs in the NtrC AAA+ domain are required for growth in defined medium. (A) Terminal OD_{660} of WT, Δ*ntrC*, *ntrC*^{K178A} (Walker A mutant), and *ntrC*^{D235A} (Walker B mutant) after 24 hours (h) of growth in M2G. Data represent mean ± standard deviation of three independent replicates.

bioRxiv preprint doi: https://doi.org/10.1101/2023.06.06.543975; this version posted August 31, 2023. The copyright holder for this preprint (which was not certified by peer review) is the author/funder, who has granted bioRxiv a license to display the preprint in perpetuity. It is made available under aCC-BY 4.0 International license.

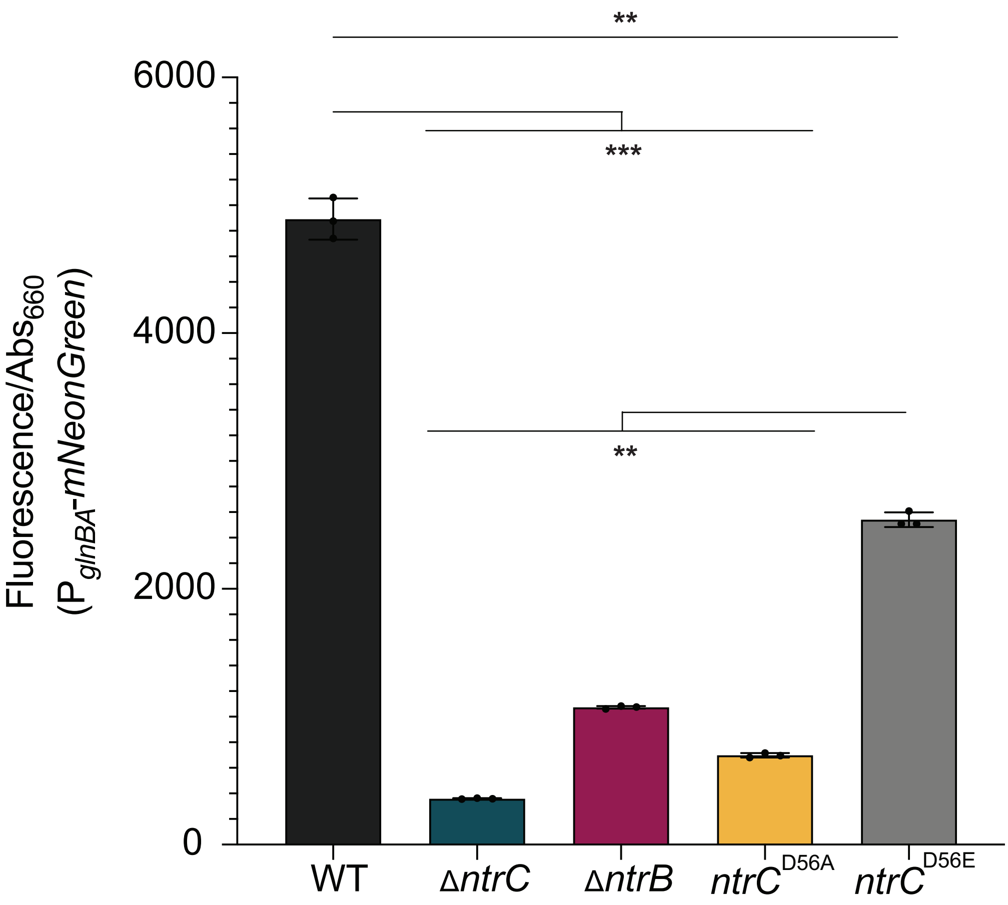

Figure S4. P$_{glnBA}$ transcription is diminished in *ntrB* and *ntrC* mutant strains. Transcription from the *glnBA* promoter (P$_{glnBA}$) was measured in WT, Δ*ntrC*, Δ*ntrB*, *ntrC*^{D56A}, *ntrC*^{D56E} using a P$_{glnBA}$-*mNeonGreen* transcriptional fusion reporter. Strains were grown to stationary phase in PYE. Fluorescence signal was measured and normalized to OD_{660}. Data represent mean ± standard deviation of three replicates. Statistical significance was determined by one-way ANOVA followed by Tukey's multiple comparisons test (*** $P < .0001$, ** $P \leq .0008$).

bioRxiv preprint doi: https://doi.org/10.1101/2023.06.06.543975; this version posted August 31, 2023. The copyright holder for this preprint (which was not certified by peer review) is the author/funder, who has granted bioRxiv a license to display the preprint in perpetuity. It is made available under aCC-BY 4.0 International license.

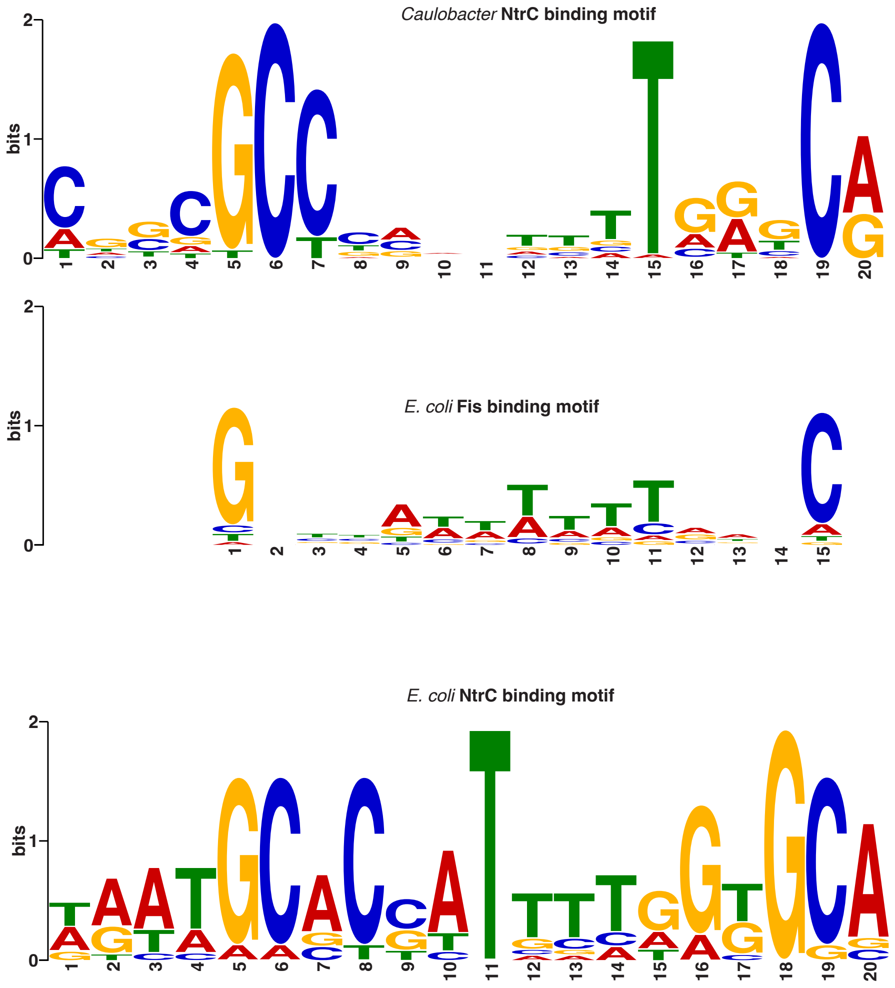

Figure S5. *Caulobacter* NtrC binding motif is similar to *E. coli* Fis and NtrC binding motifs. A search of prokaryotic transcription factor binding motifs in SwissRegulon revealed significant similarity between the *Caulobacter* NtrC binding motif and the Fis and NtrC binding motifs of *E. coli* ($P < 0.001$).

bioRxiv preprint doi: https://doi.org/10.1101/2023.06.06.543975; this version posted August 31, 2023. The copyright holder for this preprint (which was not certified by peer review) is the author/funder, who has granted bioRxiv a license to display the preprint in perpetuity. It is made available under aCC-BY 4.0 International license.

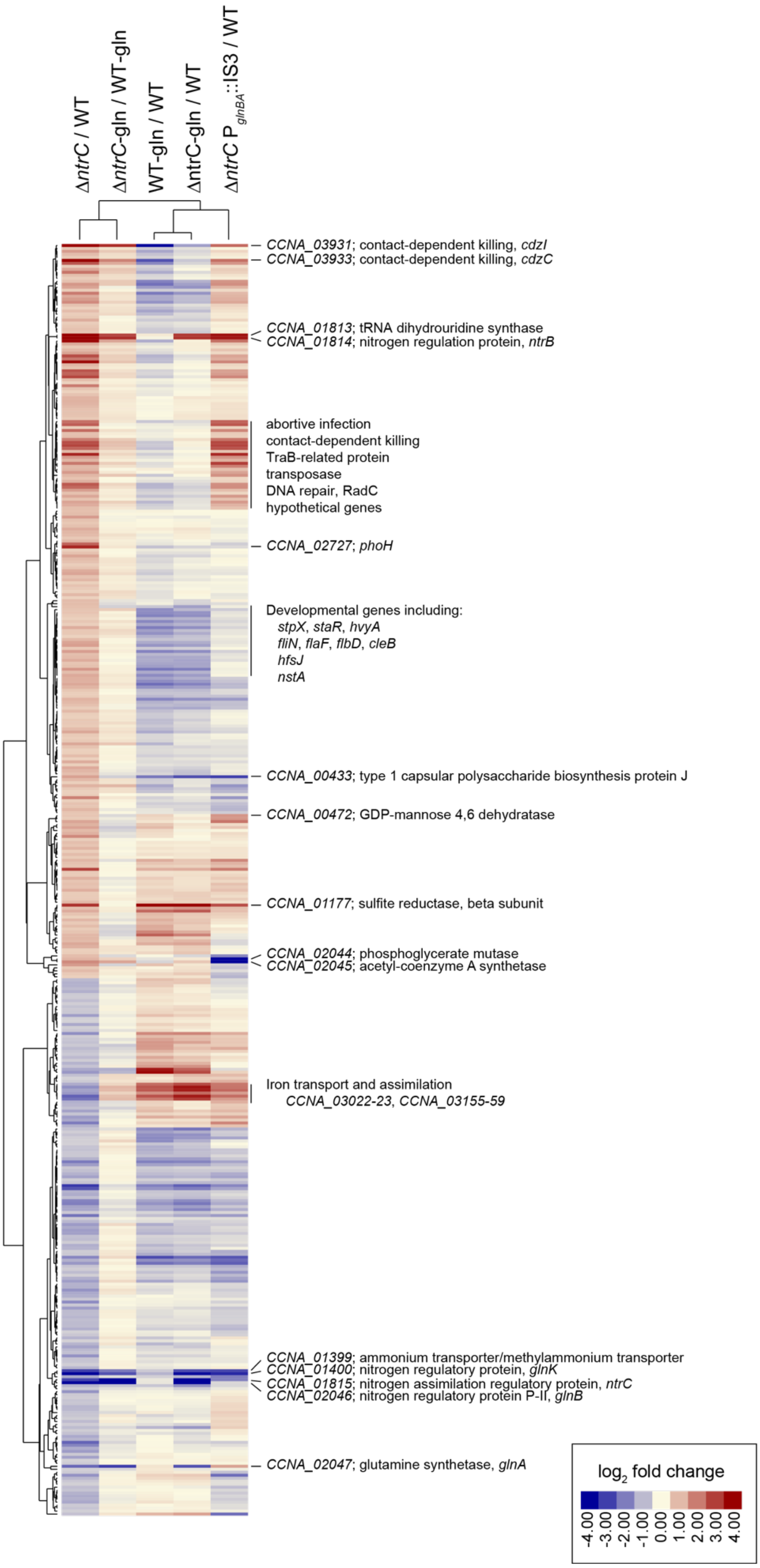

Figure S6. Nitrogen-dependent regulation of the NtrC regulon revealed by RNA-seq analysis. The heat map displays the $\log_2$ fold change of 473 genes differentially expressed between the Δ*ntrC* mutant and WT. Genes with fold change > 1.5, FDR $P < 10^{-6}$, and WT CPM > 10 are included. Each row represents a gene. Each column represents a comparison between strains and/or different media conditions (PYE complex medium and PYE supplemented with 9.3

bioRxiv preprint doi: https://doi.org/10.1101/2023.06.06.543975; this version posted August 31, 2023. The copyright holder for this preprint (which was not certified by peer review) is the author/funder, who has granted bioRxiv a license to display the preprint in perpetuity. It is made available under aCC-BY 4.0 International license.

mM glutamine (gln)). Hierarchical clustering using Cluster 3.0 was applied, employing an uncentered correlation similarity metric and average linkage for grouping.

bioRxiv preprint doi: https://doi.org/10.1101/2023.06.06.543975; this version posted August 31, 2023. The copyright holder for this preprint (which was not certified by peer review) is the author/funder, who has granted bioRxiv a license to display the preprint in perpetuity. It is made available under aCC-BY 4.0 International license.

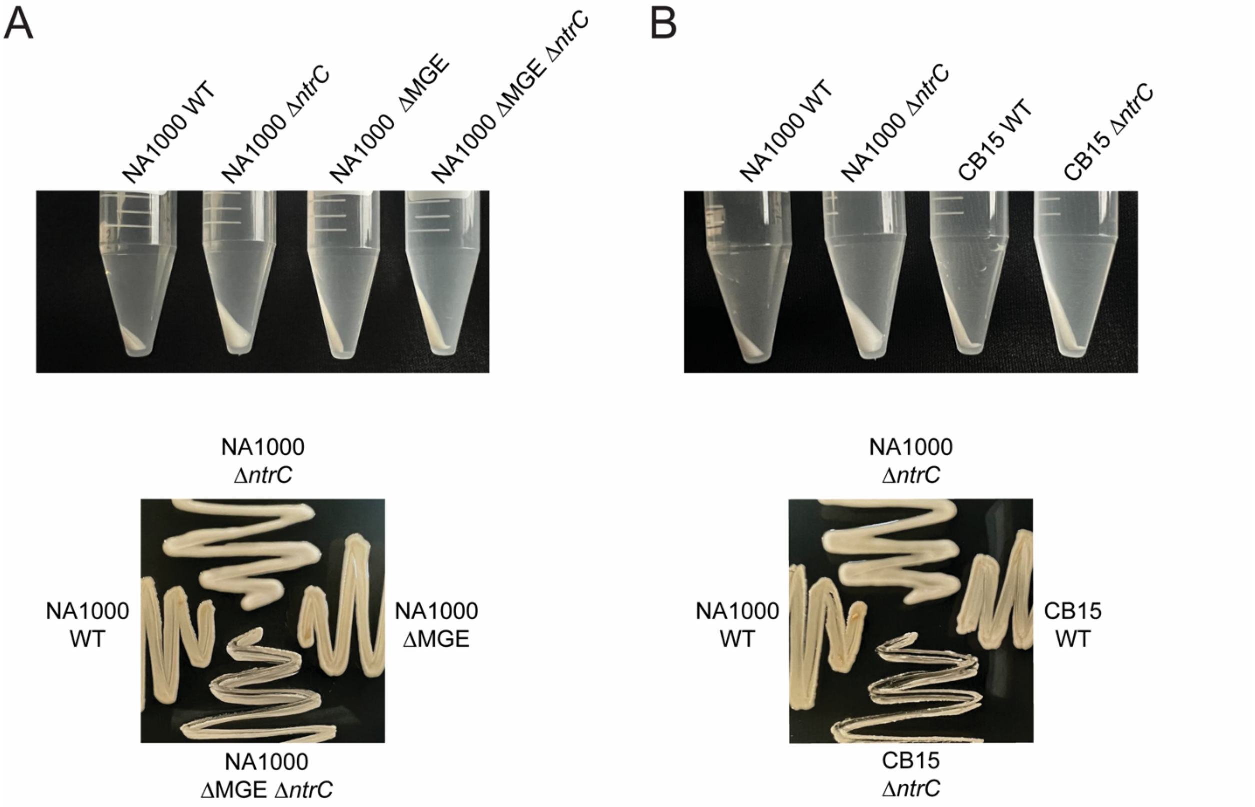

Figure S7. Mucoid phenotype of Δ*ntrC* requires the presence of the 26 kb mobile genetic element (MGE). (A) Top panel: Cell pellets of *Caulobacter crescentus* strain NA1000 WT, NA1000 Δ*ntrC*, NA1000 in which the MGE had spontaneously excised (NA1000 ΔMGE), and NA1000 ΔMGE Δ*ntrC*. Strains were grown overnight in PYE. Overnight cultures were normalized to OD_{660} = 0.5 and cells from 10 ml were centrifuged at 7,197 x g for 3 min at 4°C. Bottom panel: Growth of NA1000 WT, NA1000 Δ*ntrC*, NA1000 ΔMGE Δ*ntrC* on PYE agar supplemented with 3% sucrose. Plates were incubated for 4 days at 30°C. (B) Top panel: Cell pellets of NA1000 WT, NA1000 Δ*ntrC*, CB15 WT, and CB15 Δ*ntrC*. Cell pellets were prepared as described in panel A. Bottom panel: Growth of NA1000 WT, NA1000 Δ*ntrC*, CB15 WT, and CB15 Δ*ntrC* in same conditions described in panel A.
